# Indigenous peoples, tobacco use and the role of the commercial tobacco industry

**DOI:** 10.1111/resp.14869

**Published:** 2024-12-10

**Authors:** Raglan Maddox, Lisa J. Whop

**Affiliations:** ^1^ Bagumani (Modewa) Clan Milne Bay Papua New Guinea; ^2^ Yardhura Walani. National Centre for Aboriginal and Torres Strait Islander Wellbeing Research, National Centre for Epidemiology and Population Health, Research School of Population Health The Australian National University Canberra Australian Capital Territory Australia; ^3^ Wagadagam, Gumulgal Torres Strait Queensland Australia

**Keywords:** indigenous, smoking cessation, tobacco, tobacco control, tobacco industry

The commercial Tobacco and Nicotine Industry commonly opposes and undermines initiatives aimed at reducing and eliminating commercial tobacco use, disease and death among Indigenous peoples and the general population.^1^, ^2^, ^3^, ^4^ This includes actively promoting tobacco use to Indigenous peoples and the general population through predatory advertising, misleading health claims, and sponsorships.^1^, ^2^, ^5^ These activities attempt to normalize and glamorize nicotine addiction and tobacco use, which inadvertently seep into our everyday vernacular (e.g., ‘smoko’) and behaviours.^6^

Advertising has appropriated and used Indigenous imagery targeted toward Indigenous peoples.^1^, ^2^, ^3^, ^4^ In the United States, R.J. Reynolds markets and profits from selling Natural American Spirit (Figure 1A), which uses Indigenous imagery and misleading packaging, creating false perceptions that ‘organic’ or ‘natural’ cigarettes are less harmful.^7^, ^8^ In the 1980s, tobacco company WD & HO Wills ran advertising in Australia with the slogan ‘Get your own black’.^3^ In the 1990s, Winfield advertisements depicted an Aboriginal man playing a didgeridoo with the slogan ‘Australians' answer to the peace pipe’.^3^ More recently, Philip Morris International (PMI) sold cigarettes in Israel labelled ‘Māori Mix’^3^ and contacted Aboriginal organizations to promote e‐cigarettes (Figure 1B).^1^, ^2^ Additionally, the Centre for Research Excellence: Indigenous Sovereignty and Smoking (COREISS) was established with funding from the PMI‐funded Foundation for a Smokefree World funded.^2^, ^9^ COREISS has opposed public health initiatives, including the Smokefree Aotearoa 2025 Action Plan and restrictions on smoking in cars with children.^9^ The Director of COREISS addressed the New Zealand Health Select Committee regarding proposed legislation to ban smoking in cars with children in 2019, stating:

‘*That's junk science that the toxins are first of all harmful and that they build up. As I said there's no evidence that nicotine or the particles in cigarette smoke, the residue delivers any ill health to anybody … obviously it's damaging for the person who's smoking. But ah, it's, as I say in my submission, the dose makes the poison. Children are not in the car all day long every day*’.

‘…*what gets missed is that people don't realise that our bodies heal, so even if we are temporarily exposed, we heal from that*’^9^, ^10^

**FIGURE 1:**
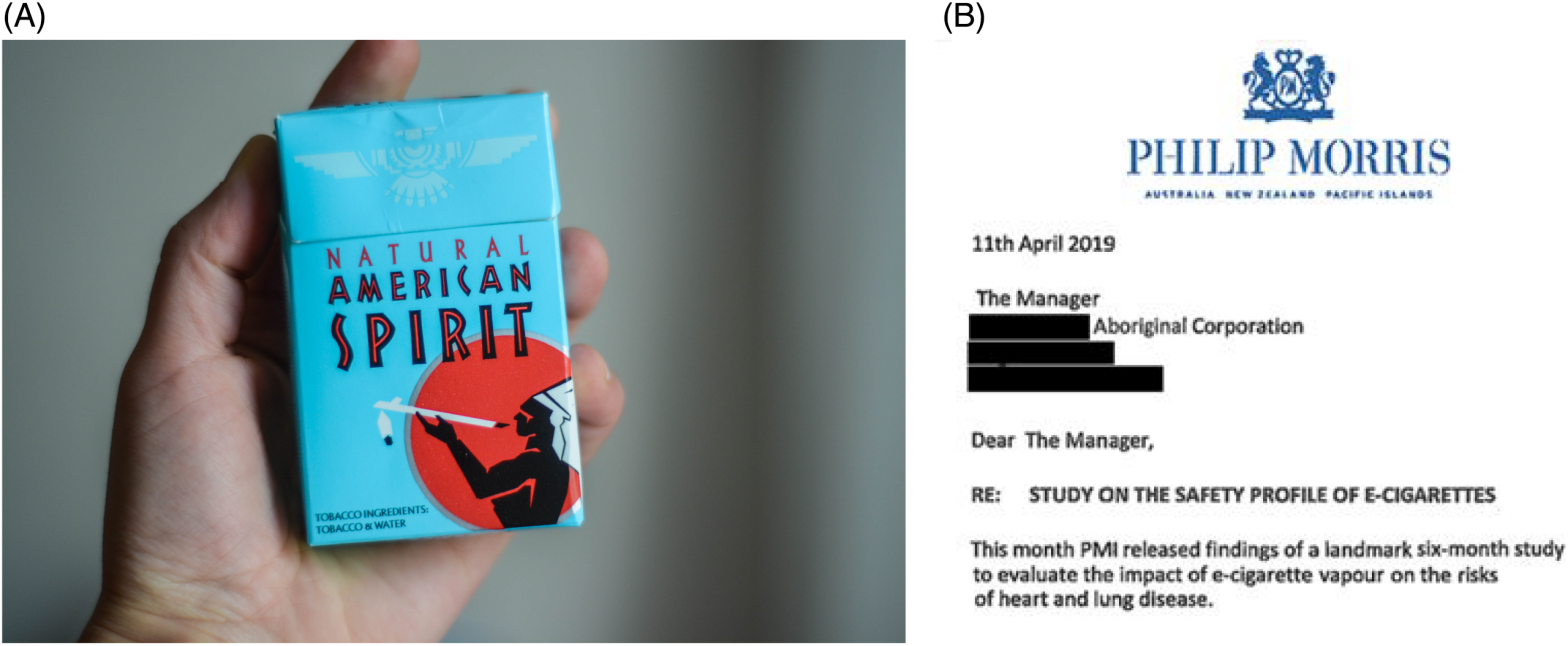
(A) Natural American Spirit packet of cigarettes (*Source*: Olesea Vetrila/Shutterstock.com); (B) Letter from the Tobacco Industry targeting Indigenous organizations (reproduced with permission from Maddox et al.^1^).

In November 2019, Jeff Gaulin, the Director of External Affairs at Rothmans, Benson & Hedges Inc., opened a harm reduction conference in Canada with the question, ‘So why is the world's largest maker of cigarettes assembling a harm reduction conference?’ This question is particularly notable, as it highlights the ways in which the Tobacco Industry often positions itself as having ‘good intentions’, while simultaneously obfuscating its true motives and involvement. Notably, Rothmans, Benson & Hedges Inc. was absent from the conference's promotional materials, leaving attendees unaware of their role. It was only when Gaulin explicitly identified himself as a key organizer that the link between conference funding and the Tobacco Industry became apparent. Such examples help to illustrate a range of mechanisms used in the Tobacco and Nicotine Industry toolkit, with strong evidence that Indigenous people are disproportionately impacted by industry‐generated harms.^11^, ^12^, ^13^

Early settlers used tobacco as an incentive to achieve their goals, encouraging trade and embedding tobacco use across society.^14^ Up to the late 1960s, Aboriginal and Torres Strait Islander peoples were paid with rations of tobacco in lieu of wages, excluding Indigenous peoples from the cash economy and education system and systematically entrenching tobacco use and nicotine addiction.^11^, ^14^ Smoking causes over 20,000 deaths each year across Australia,^15^ including 37% of all Aboriginal and Torres Strait Islander deaths and 50% of Aboriginal and Torres Strait Islander deaths for those over 45 years old.^16^ Understanding this context can help avoid false stereotypes, such as the assertion that Indigenous peoples are more likely to smoke due to biological inferiority.^17^, ^18^

The commercial Tobacco and Nicotine Industry continues to try to exploit Indigenous peoples by manufacturing and promoting highly addictive and harmful products.^3^, ^6^, ^18^, ^19^ The Tobacco and Nicotine Industry generates a narrative of nicotine addiction as a ‘personal choice’ that supposedly provides freedom, liberation and power.^6^, ^20^ This carefully curated narrative shifts blame onto individuals for using commercial tobacco, leading to stigma and discrimination, including suboptimal experiences within the healthcare system.^6^, ^21^ This is particularly harmful because most people start smoking at a young age without fully understanding the lifelong implications of addiction.^22^, ^23^

Companies are using advances in data and technology to target people based on age, gender, income and where they live. For example, algorithms are used to tailor marketing strategies and increase availability of nicotine products according to area demographics and shops are tactically opened near schools and in low‐income areas.^24^, ^25^ Social media and technology is also used to target younger generations with the evolution of e‐cigarettes/vapes.^26^ Similar to tobacco use, vaping disproportionately affects Indigenous peoples in countries with ongoing colonial histories.^27^ Daily vaping rates among Māori (Indigenous peoples of Aotearoa New Zealand) are almost three times higher than people of European descent (Māori: 23.5%; European: 8.3%) with similar trends among Year 10 students: 22.3% of Māori youth use e‐cigarettes daily compared to 7.5% of European descent students.^28^ Further, a recent study in Australia found approximately 80% of 14–17‐year‐olds found it easy to access vapes,^29^ and people who vape are three times more likely to go on to smoke than those who do not vape.^30^

Structural changes are required to disrupt, reduce and eradicate commercial tobacco and nicotine related disease and death.^12^, ^19^, ^31^ This includes logical, evidence‐based structural measures to (1) reduce the number of retail outlets, (2) regulate tobacco and nicotine products to make them less addictive and (3) phase out the legal sale of tobacco by initiating a nicotine‐free generation that can help level the nicotine addiction and dependence playing field.^19^, ^31^ These structural reforms can help to prevent and address addiction, supporting the fact that most Aboriginal and Torres Strait Islander and Māori peoples who smoke want to quit or wish they had never started.^13^, ^32^

Healthcare practitioners must ensure that every client who smokes and/or vapes is offered support to quit as part of their everyday care, consistent with the World Health Organization's Framework Convention on Tobacco Control.^12^ This includes doctors and healthcare practitioners providing cessation supports, including pharmacotherapy where appropriate.^12^ The crucial role of health professionals in supporting quit attempts is well‐established.^12^, ^33^ However, the routine and consistent provision of cessation supports has been lacking across all healthcare settings, indicating substantial room for improvement. Barriers such as time constraints, costs and practitioner knowledge need to be addressed. Overcoming these obstacles and integrating cessation support can help uphold the Human Right to Health. Additionally, it is essential to understand the context of nicotine addiction and recognize the extensive efforts and significant expenditure by the commercial Tobacco and Nicotine Industry to undermine this right. The industry has ensured that a variety of highly addictive tobacco and nicotine products, such as vapes are easily accessible, thereby normalizing and glamorizing their use. This makes quitting extremely challenging.

Quitting is never easy, but with a range of support programs outlined in the National Tobacco Strategy, along with the upcoming national lung cancer screening program and the Tackling Indigenous Smoking program across Australia, significant improvements in health outcomes are achievable.^14^, ^33^, ^34^ The Tackling Indigenous Smoking Program is an Aboriginal and Torres Strait Islander‐led public health initiative. This is designed to implement locally tailored health promotion activities to prevent the uptake of smoking and vaping, promote quitting and encourage referrals to cessation services.^14^

Urgent national action is imperative to counter the relentless efforts of the commercial Tobacco and Nicotine Industry, which perpetuates addiction and profits at the expense of public health, especially among Indigenous communities. What is needed is decisive leadership committed to rapid, whole‐population impact. Consistent with Australia's National Tobacco Strategy 2023–2030, essential structural reforms, including reducing retail outlets and regulating tobacco products, such as minimizing nicotine content to the lowest possible level.^19^

Addressing the product itself is critical—nicotine‐free or ultralow‐nicotine products can help prevent uptake, facilitate cessation and prevent relapse, aligning with Priority 7.6 of Australia's National Tobacco Strategy 2023–2030. Additionally, track and trace technology should also be implemented to help identify and control illicit trade, particularly in communities that have chosen to be tobacco and nicotine‐free.

Finally, all healthcare practitioners, including those in respiratory care, must prioritize and consistently deliver culturally safe cessation supports at every opportunity.^14^ This commitment will improve health outcomes and help to address a completely preventable cause of death and disease among Aboriginal and Torres Strait Islander peoples, and all Australians.

## CONFLICT OF INTEREST STATEMENT

None declared.
